# Association between CD56, CD38 and chronic endometritis and analysis of pregnancy prognosis: a single-center retrospective cohort study

**DOI:** 10.3389/fmed.2026.1723552

**Published:** 2026-02-11

**Authors:** Yanni Kong, Shiwei Zhou, Qianru Zhou

**Affiliations:** 1 Department of Traditional Chinese Medicine, Center for Reproductive Medicine, Zhejiang Provincial People’s Hospital (Affiliated People’s Hospital), Hangzhou Medical College, Hangzhou, Zhejiang, China; 2 Department of Pediatrics, Hangzhou TCM Hospital Affiliated to Zhejiang Chinese Medical University, Hangzhou, Zhejiang, China

**Keywords:** chronic endometritis, pregnancy prognosis, CD38, CD56, cohort study

## Abstract

**Background:**

The clinical symptoms of chronic endometritis (CE) are atypical, but it is correlated with adverse pregnancy outcomes. The accuracy of its detection needs to be further improved. This article aims to study the association between immunohistochemical CD56, CD38, and CE, and to explore their effect on pregnancy outcomes.

**Methods:**

A retrospective analysis was conducted on the clinical data of 844 patients who underwent hysteroscopy check/surgery in the Department of Traditional Chinese Medicine of Zhejiang Provincial People’s Hospital from May 1, 2023 to October 31, 2024. The expression levels of CD56 and CD38 and the 12-week continuous pregnancy rate were compared between the CE group and the non-CE group. The optimal cut-off values of CD56 and CD38 were taken based on the ROC curve to explore their diagnosis value for CE and predictive value for pregnancy outcomes.

**Results:**

Among the 844 patients, the levels of CD56 and CD38 in the CE group were both higher than those in the non-CE group (*P* < 0.05). Spearman correlation analysis indicated that CD56 and CD138, CD38, and CD138, and CD56 and CD38 were correlated, respectively (*r* = 0.18, *r* = 0.38, *r* = 0.33, *P* < 0.001 for all). The optimal cut-off value was selected based on the ROC curve. The incidence of chronic endometritis was higher in the high-value CD56 group (≥ 23.5 cells/HPF), and in the high-value CD38 group (≥ 0.5 cells/HPF) (χ^2^ = 15.994, χ^2^ = 129.067, and both *P* < 0.001). However, there were no statistically significant differences in the 12-week continuous pregnancy rates between the CE group and the non-CE group, the high-value CD56 group and the low-value CD56 group, and the high-value CD38 group and the low-value CD38 group (*P* > 0.05). Taking the pregnancy outcome as the predictive target and re-taking the optimal cutoff value, the 12-week continuous pregnancy rate of the new high-value CD38 group (≥ 9.5 cells/HPF) was lower than that of the new low-value CD38 group (< 9.5 cells/HPF) (χ^2^ = 5.504, *P* = 0.019), and the difference was statistically significant.

**Conclusion:**

CD56 and CD38 are associated with CE. Various immunohistochemicals can be utilized to improve the accuracy of CE diagnosis, and CD38 is better for evaluating pregnancy outcomes.

## 1 Introduction

Chronic endometritis (CE) is a persistent localized inflammatory disease of the endometrium. The main pathological features are endometrial surface edema, increased density of stromal cells, isolation and maturation of epithelial cells and stromal fibroblasts, and infiltration of endometrial stromal plasma cells (ESPCs) (1, 2). The clinical symptoms are often atypical or only accompanied by mild lower abdominal discomfort, increased vaginal discharge, abnormal uterine bleeding, painful sexual intercourse and other symptoms (3, 4). In recent years, the detection rate of CE in patients with infertility, recurrent spontaneous abortion (RSA) and repeated implantation failure (RIF) has been continuously increasing (5–8). Studies have shown that CE may affect the endometrial microecology, induce abnormal expression of immune cells and cytokines, mediate inflammatory responses, and reduce endometrial receptivity (9, 10), thereby influencing pregnancy outcomes. However, the standardized definition and general diagnostic criteria for CE have not yet been unified and clarified. Currently, the diagnostic methods for CE mainly include hysteroscopy and pathological biopsy. Cicinelli proposed the diagnostic criteria for CE at fluid hysteroscopy (11): (A) strawberry aspect: large areas of hyperemic endometrium flushed with white central points; (B) focal hyperemia: small areas of hyperemic endometrium; (C) hemorrhagic spots: focal red areas with sharp and irregular borders possibly in continuity with capillary; (D, E) focal or diffuse micropolyps: small intrauterine new growths < 1 mm in size with a distinct connective-vascular axis, distributed on focal areas (D) or on all of the endometrial surface (E); (F) thick and pale appearance of the endometrium in the follicular phase owing to stromal edema (a normal finding during the secretory phase). In addition, the currently recognized pathological diagnostic criteria for CE mainly focus on ESPCs infiltration. CD138, as the immunohistochemical molecule with the highest specificity for recognizing plasma cells, has better objectivity and accuracy (12, 13), and has been widely applied and has become the preferred method for diagnosing CE. CD38 is also a common marker on the surface of plasma cells. Although its specificity is not as good as CD138, some studies suggest that it is more sensitive (14). CD56 is mainly a marker on the surface of natural killer cells (NK cells) which are related to pregnancy outcomes. Our team has found in clinical practice that the changing trends of CD56, CD38, and CD138 are often consistent. This study aims to explore the association between CD56, CD138 and chronic endometritis as well as their predictive role in pregnancy outcomes, with the expectation of providing certain reference and guidance for clinical practice. This study was approved by the Medical Ethics Committee of Zhejiang Provincial People’s Hospital.

## 2 Materials and methods

### 2.1 Research object

A retrospective analysis was conducted on the clinical data of patients who underwent hysteroscopy check/surgery in the Department of Traditional Chinese Medicine of Zhejiang Provincial People’s Hospital from May 1, 2023 to October 31, 2024. Inclusion criteria: age 20–40 years old; undergo standard hysteroscopy check/surgery and complete endometrial pathological biopsy; pathological tissues underwent immunohistochemical tests for CD138, CD56 and CD38; the clinical case data are complete. Exclusion criteria: combined with acute or subacute reproductive tract infections; those with atypical endometrial hyperplasia or endometrial cancer; those with other serious chronic diseases, such as cardiovascular and cerebrovascular diseases, liver and kidney dysfunction, mental disorders, etc., those with incomplete clinical data.

### 2.2 Research methods

All patients underwent hysteroscopy check/surgery within 7 days after the end of menstruation. During the operation, a comprehensive hysteroscopy was used to examine the uterine cavity. Based on the actual condition of the patients in the uterus, corresponding surgeries were performed and endometrial biopsy was conducted. Hysteroscopic examination/surgery is jointly performed and evaluated by two doctors (one of whom is a senior chief physician) under hysteroscopy. The collected tissue samples were subjected to conventional dewaxing and hydration, then HE staining was performed and observed under a microscope. At the same time, standardized immunohistochemical tests were conducted to observe and record the expression of CD138, CD56, and CD38. After rechecking all the causes of the disease by two pathologists, a report was issued.

### 2.3 Research groups and observation indicators

A total of 844 patients were included in this study. Taking positive CD138 as the basis for diagnosing CE, there were 495 cases in the CE group and 349 cases in the non-CE group. The levels of CD56 and CD38 in the two groups were compared. Excluding 162 cases that underwent hysteroscopic reexamination (The reexamination is scheduled for the following month, that is, within 7 days after the end of menstruation in the next month), 57 cases with no fertility needs, and 281 cases that did not have regular outpatient follow-up 1 year after the operation and had unknown pregnancy outcomes, a total of 344 cases were included for pregnancy outcome analysis (12-week continuous pregnancy rate = number of successful pregnancies at 12 weeks/total number of cases × 100%). Demographic table for sampled patients is shown in Table 1.

**TABLE 1:** Demographic table.

Group	Number	Age	Pregnancy outcomes	
			Pregnancy	Non-pregnancy
CE group	349	32.12 ± 0.208	85	48
Non-CE group	495	32.07 ± 0.179	150	61
Total	844		235	109

### 2.4 Statistical analysis

Data processing was carried out using SPSS 25.0 software. After the Shapiro-Wilk test, when the measurement data conformed to the normal distribution, they were expressed as mean ± standard deviation (± s), and the independent sample *t*-test was used for comparison between groups. When the normal distribution was not followed, the median (P25, P75) was used, and the Mann-Whitney U rank sum test was used for comparison between groups. Counting data are denoted by n and the chi-square test is used. The predictive value test was conducted using the ROC curve, and the optimal cut-off value was determined based on the Youden index. Spearman correlation analysis and paired plotting were performed using R version 4.5.1. *P* < 0.05 was considered statistically significant.

## 3 Results

### 3.1 Comparison of CD56, CD38 and pregnancy outcomes between the CE group and the non-CE group

As shown in Table 2, there were significant differences in the levels of CD56 and CD38 between the two groups of patients. The levels of CD56 and CD38 in the CE group were higher than those in the non-CE group, and the differences were statistically significant (*P* < 0.05). As shown in Table 3, there was no statistically significant comparison of the 12-week continuous pregnancy rate between the CE group and the non-CE group (*P* > 0.05).

**TABLE 2:** Comparison of CD56 and CD38 levels between the CE group and the non-CE group.

Group	CD56	CD38
CE group (*n* = 349)	30 (20,50)	9 (4,18)
Non-CE group (*n* = 495)	30 (18,41.5)	3 (0,10)
*z-*value	-4.439	-10.131
*P-*value	< 0.001	<0.001

**TABLE 3:** Comparison of pregnancy outcomes between the CE group and the non-CE group.

Group	Pregnancy	Non-pregnancy	χ ^2^ value	*P-*value
CE group (*n* = 133)	85 (63.91%)	48 (36.09%)	1.943	0.163
Non-CE group (*n* = 211)	150 (71.09%)	61 (28.91%)		

### 3.2 Comparison of CE incidence and pregnancy outcomes between the high and low value groups of CD56 and CD38

According to the ROC curve, taking the CD56 and CD38 values corresponding to the maximum Youdon index as the cut-off values, the patients were divided into high and low value groups. The ROC curve showed that the area under the curve (AUC) of CD56 and CD38 for predicting chronic endometritis was 0.589 and 0.703, and the optimal cut-off values were 23.5 and 0.5. As shown in Table 4, the incidence of CE was higher in the high-value CD56 group (≥ 23.5 cells/HPF) than in the low-value CD56 group (< 23.5 cells/HPF) (χ^2^ = 15.994, *P* < 0.001). The incidence of CE in the high-value CD38 group (≥ 0.5 cells/HPF) was higher than that in the low-value CD38 group (< 0.5 cells/HPF) (χ^2^ = 129.067, *P* < 0.001), and the differences were statistically significant. However, as shown in Table 5, there was no statistically significant difference in the 12-week continuous pregnancy rate between the high-value and the low-value CD56 and CD38 groups (*P* > 0.05).

**TABLE 4:** Comparison of the incidence of CE in the high and low value groups of CD56 and CD38.

Name	Group	CD138(+)	CD138(-)	χ ^2^ value	*P*-value
CD56	High-value group (*n* = 533)	248 (46.53%)	285 (53.47%)	15.994	< 0.001
	Low-value group (*n* = 311)	101 (32.48%)	210 (67.52%)		
CD38	High-value group (*n* = 646)	336 (52.01%)	310 (47.99%)	129.067	< 0.001
	Low-value group (*n* = 198)	13 (6.57%)	185 (93.43%)		

**TABLE 5:** Comparison of pregnancy outcomes between the high and low CD56 and CD38 groups.

Name	Group	Pregnancy	Non-pregnancy	χ ^2^ value	*P*-value
CD56	High-value group (*n* = 215)	145 (67.44%)	70 (32.56%)	0.201	0.654
	Low-value group (*n* = 129)	90 (69.77%)	39 (30.23%)		
CD38	High-value group (*n* = 255)	172 (67.45%)	83 (32.55%)	0.339	0.560
	Low-value group (*n* = 89)	63 (70.79%)	26 (29.21%)		

According to the ROC curve, with pregnancy outcome as the predictive target, the optimal cut-off values were re-taken and divided into the new high-value group and the new low-value group. The AUC of CD56 and CD38 were 0.514 and 0.563, and the optimal cut-off values were 32.5 and 9.5 respectively. As shown in Table 6, there was still no statistically significant difference in the 12-week continuous pregnancy rate between the CD56 new high-value group (≥ 32.5 cells/HPF) and the CD56 new low-value group (< 32.5 cells/HPF) (*P* > 0.05). The 12-week continuous pregnancy rate in the new high-value CD38 group (≥ 9.5 cells/HPF) was significantly lower than that in the new low-value CD38 group (< 9.5 cells/HPF) (χ^2^ = 5.504, *P* = 0.019).

**TABLE 6:** Comparison of pregnancy outcomes between the new high and low value groups of CD56 and CD38.

Name	Group	Pregnancy	Non-pregnancy	χ ^2^ value	*P*-value
CD56	New high-value group (*n* = 142)	92 (64.79%)	50 (35.21%)	1.388	0.239
	New low-value group (*n* = 202)	143 (70.79%)	59 (29.21%)		
CD38	New high-value group (*n* = 118)	71 (60.17%)	47 (39.83%)	5.504	0.019
	New low-value group (*n* = 226)	164 (72.57%)	62 (27.43%)		

### 3.3 Correlation analysis among CD138, CD56, and CD38

**FIGURE 1:**
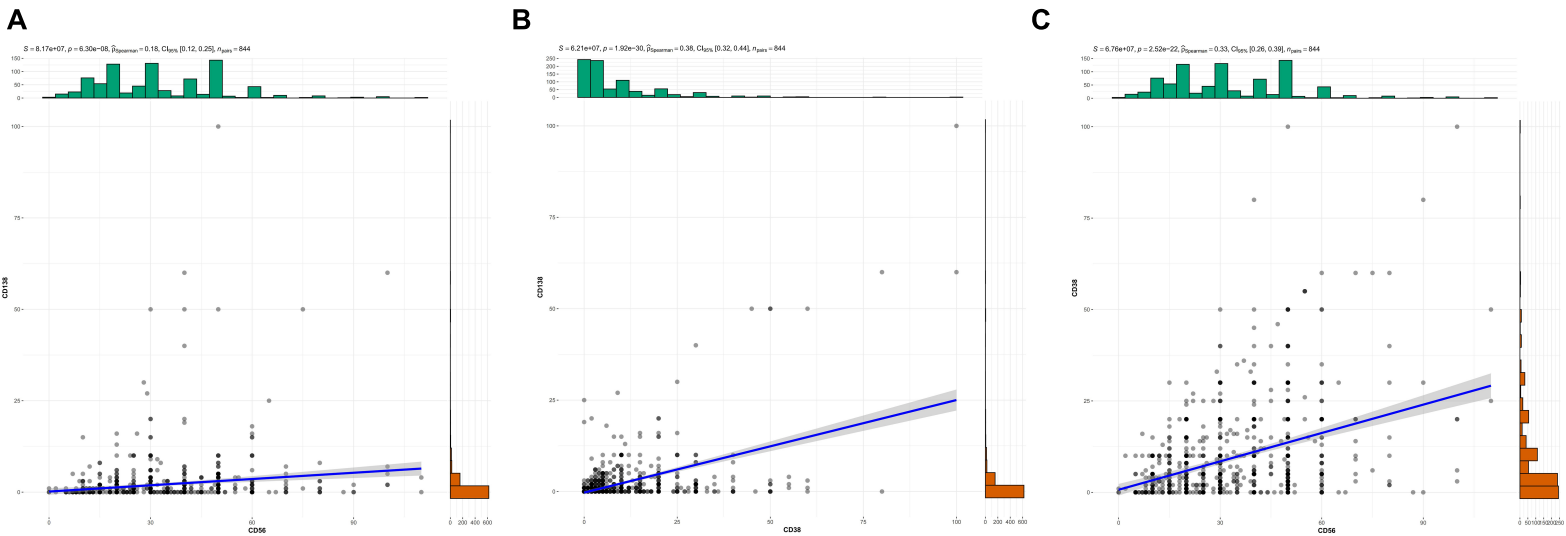
The correlation among CD138, CD56, and CD38. **(a)** Correlation between CD56 and CD138. **(b)** Correlation between CD38 and CD138. **(c)** Correlation between CD56 and CD38.

Spearman correlation analysis indicated that CD56 was positively correlated with CD138 (*r* = 0.18, *P* < 0.001), CD38 was positively correlated with CD138 (*r* = 0.38, *P* < 0.001), and CD56 was positively correlated with CD38 (*r* = 0.33, *P* < 0.001) (Figure 1).

### 3.4 The pairing results of CD138, CD56, and CD38 before and after the recheck

**FIGURE 2:**
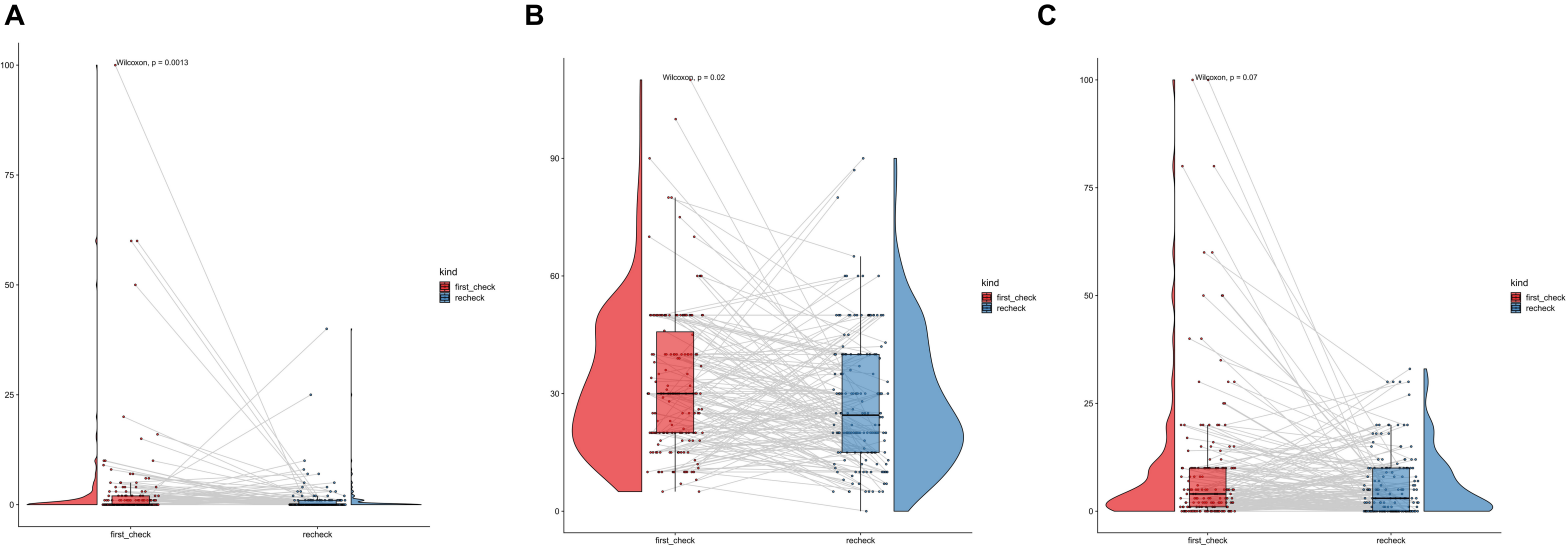
The pairing results of CD138, CD56, and CD38 before and after the recheck. **(a)** Pairing results of CD138. **(b)** Pairing results of CD56. **(c)** Pairing results of CD38.

The pairing results of 162 patients before and after the recheck showed that the levels of CD138 and CD56 after recheck were both lower than before, and the differences were statistically significant (*P* = 0.0013, *P* = 0.02). There was no statistically significant difference in CD38 before and after recheck (*P* > 0.05) (Figure 2).

## 4 Discussion

### 4.1 CD38 and CD56 are correlated with chronic endometritis

The traditional diagnosis of CE is to identify plasma cells through hematoxylin-eosin (HE) staining of endometrial biopsy tissues, but sometimes it is difficult to distinguish and it is easy to cause misdiagnosis or missed diagnosis. CD138 and CD38 are common markers on the surface of plasma cells. Among them, CD138 is a specific marker that can label plasma cells including spindle-shaped plasma cells (15), while CD38 has strong coloring and more obvious characteristics, which can improve the accuracy of plasma cell identification (16). CD56 is mainly a marker on the surface of NK cells. Uterine natural killer cells (uNK cells) are a special subset of NK cells existing in the endometrium. During the normal proliferation period, most uNK cells are inactive. However, in the late luteal phase and early pregnancy, uNK cells can proliferate in large quantities. They are not highly cytotoxic and their main function is to secrete various cytokines, thereby promoting vascular remodeling, trophoblast invasion and placental development (17, 18), which is crucial for successful pregnancy. In the endometrium of CE patients, the number of CD56 + uNK cells is significantly higher than the normal level, disrupting the local immune homeostasis. Moreover, the inflammatory environment may activate these uNK cells to produce more pro-inflammatory factors and exhibit stronger cytotoxicity (19, 20), causing their function to shift from “immune nutrition” to “immune attack.” This can cause a rejection reaction to the embryo, leading to failed implantation or early miscarriage. For 844 sampled patients in this study, the rank sum test results showed that the CD56 and CD38 in the CE group were both higher than those in the non-CE group, and the difference was statistically significant (*P* < 0.05). Spearman correlation analysis indicated that there was a positive correlation between CD56 and CD138, CD38, and CD138, and CD56 and CD38 (*r* = 0.18, *r* = 0.38, *r* = 0.33, *P* < 0.001 for all). Furthermore, studies have shown that MUM-1 and CD138 have similar accuracy in CE diagnosis. Moreover, compared with CD138, MUM-1 is particularly useful for identifying plasma cells when they are scarce or poorly differentiated, and it demonstrates higher inter-observer consistency than other markers (21). Therefore, in clinical practice, a comprehensive assessment can be conducted by combining the manifestations under hysteroscopy and the detections of CD138, CD38, CD56, MUM-1 and so on, to improve the accuracy of the CE detection rate.

### 4.2 What are the optimal cut-off values for CD38 and CD56 in the diagnosis of CE?

Since CD38 and CD56 levels correlated with chronic endometritis (CE), we used ROC curves to define diagnostically significant thresholds. The optimal cut-off values for predicting CE were 23.5 for CD56 and 0.5 for CD38. The chi-square test results indicated that the incidence of CE was significantly higher in patients with CD56 ≥ 23.5 cells/HPF compared to those with CD56 < 23.5 cells/HPF, and similarly higher in patients with CD38 ≥ 0.5 cells/HPF compared to those with CD38 < 0.5 cells/HPF (χ^2^ = 15.994, χ^2^ = 129.067, both *P* < 0.001). However, CD38 expression at a threshold of ≥ 0.5 cells/HPF alone is not sufficient for the definitive diagnosis of CE. Among the 844 patients enrolled in this study, 349 were diagnosed with CE, including 336 who were CD38-positive, while 495 were non-CE patients, among whom 185 were CD38-negative. The calculated sensitivity was 96.28%, whereas the specificity was 37.37%, indicating that CD38 exhibits high sensitivity but limited specificity in detecting CE. This finding was further supported by follow-up data from 162 patients who underwent recheck. The pairing results showed that the expression levels of CD138 and CD56 after recheck were both lower than before, and the differences were statistically significant (*P* = 0.0013, *P* = 0.02), which was consistent with our common perception. In contrast, no statistically significant difference in CD38 expression was observed before and after recheck (*P* > 0.05). CD38 was initially discovered on thymocytes and T lymphocytes during the search for surface molecules involved in antigen recognition. Despite variable expression levels, CD38 is broadly expressed across the immune system and is widely recognized as an “activation marker” (22). Some studies have attempted to use the MUM-1/CD38 immunohistochemical double staining method to double-label the same glass slide (16). This method substantially enhances the intensity and accuracy of plasma cell detection, facilitates interpretation, effectively compensates for the limitations of morphological assessment, and warrants further research.

### 4.3 What is the optimal cut-off value of CD38 for predicting pregnancy outcomes?

Although CD38 exhibits high sensitivity but low specificity, making it unsuitable for the standalone diagnosis of CE, it demonstrates superior prognostic value for pregnancy outcomes compared to CD138 and CD56. Based on the ROC curve analysis, the optimal cut-off value for CD38 was determined to be 9.5 cells/HPF. The results indicated that the 12-week continuous pregnancy rate in patients with CD38 ≥ 9.5 cells/HPF was lower than in those with CD38 < 9.5 cells/HPF (χ^2^ = 5.504, *P* = 0.019), and the difference was statistically significant. CD38 may exert immunosuppressive effects through the regulation of calcium channel, thereby influencing cellular proliferation, insulin secretion, and T-cell activation. Elevated CD38 expression has been associated with increased production of pro-inflammatory cytokines and enhanced inflammatory responses (23), which may lead to the displacement or closure of the window of endometrial receptivity and consequently impair embryo implantation. Previous studies have shown that CD138 is expressed in both glandular and normal interstitial cells, whereas CD38 is negative in glandular cells and selectively expressed in interstitial plasma cells. Furthermore, CD38 demonstrates a more favorable cut-off point for predicting reproductive outcomes than CD138 (24), a finding that aligns with our results. In future research, combining multiple immunohistochemical markers may enable the development of more robust and reliable models for evaluating pregnancy outcomes.

## 5 Conclusion

In conclusion, except for the detection of endometrial CD138, the combined detection of CD56 (≥ 23.5 cells/HPF) and CD38 (≥ 0.5 cells/HPF) can further enhance the diagnostic efficiency of CE and provide a basis for clinical diagnosis and treatment. In addition, CD38 provides valuable information regarding pregnancy outcomes. When CD38 is ≥ 9.5 cells/HPF, it indicates a worse pregnancy outcome. However, this study was only a single-center retrospective study and there is a risk of false positive results due to data-driven threshold optimizationin. In the future, multi-center and large-sample prospective studies need to be conducted in order to provide patients with more definite diagnostic and treatment guidelines for CE.

## Data Availability

The original contributions presented in this study are included in this article/Supplementary material. Further inquiries can be directed to the corresponding author.
